# The chemical structures and biological activities of indole diterpenoids

**DOI:** 10.1007/s13659-022-00368-7

**Published:** 2023-01-03

**Authors:** Jingwen Niu, Jianzhao Qi, Pengchao Wang, Chengwei Liu, Jin-ming Gao

**Affiliations:** 1 Key Laboratory for Enzyme and Enzyme-Like Material Engineering of Heilongjiang, College of Life Science, Northeast Forestry University, Harbin, 150040 Heilongjiang China; 2 Shaanxi Key Laboratory of Natural Products and Chemical Biology, College of Chemistry and Pharmacy, Northwest A&F University, Yangling, 712100 Shaanxi China

**Keywords:** Indole diterpenoids, Structural classification, Physiological activity, Fungus

## Abstract

Indole diterpenoids (IDTs) are an essential class of structurally diverse fungal secondary metabolites, that generally appear to be restricted to a limited number of fungi, such as *Penicillium*, *Aspergillus*, *Claviceps*, and *Epichloe* species, etc. These compounds share a typical core structure consisting of a cyclic diterpene skeleton of geranylgeranyl diphosphate (GGPP) and an indole ring moiety derived from indole-3-glycerol phosphate (IGP). 3-geranylgeranylindole (3-GGI) is the common precursor of all IDTs. On this basis, it is modified by cyclization, oxidation, and prenylation to generate a large class of compounds with complex structures. These compounds exhibit antibacterial, anti-insect, and ion channel inhibitory activities. We summarized 204 compounds of IDTs discovered from various fungi over the past 50 years, these compounds were reclassified, and their biological activities were summarized. This review will help to understand the structural diversity of IDTs and provide help for their physiological activities.

**Graphical Abstract:**

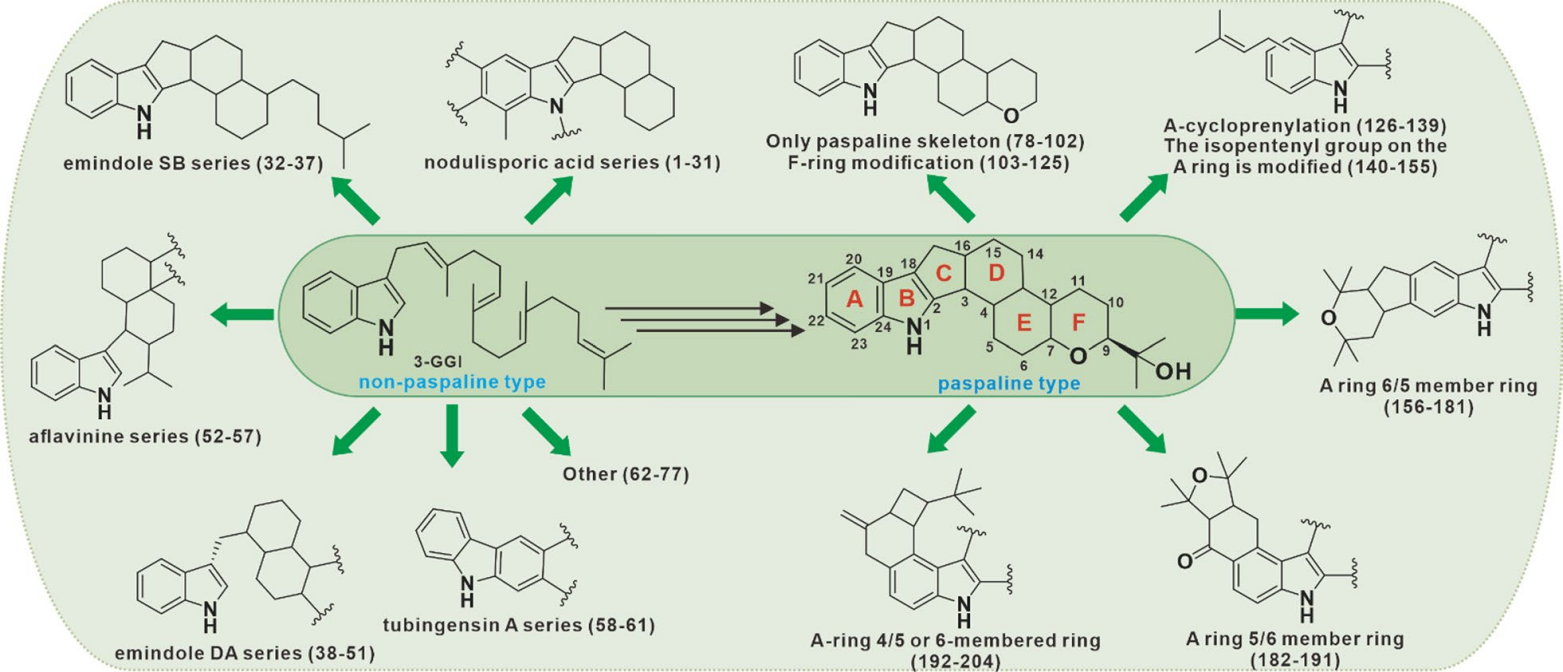

## Introduction

IDTs are a structurally diverse class of fungal secondary metabolites, all sharing a common core structure consisting of indole, and a diterpene carbon backbone derived from four mevalonate-derived isoprene units [1]. The molecular complexity of these compounds is achieved by adding more isoprene units to the core structure and by various modifications such as oxidation, cyclization, and halogenation. IDTs are ubiquitous in the natural environment, and moldy food produced by *Penicillium* sp. is a common source of these compounds. For example, the fungus *P.* *tularense* found in tomatoes has metabolites such as janthitrems, paspalinine, paxilline, etc. [2]. *P.* *crustosum*, which produces the shivering mycotoxin penitrems, is a common food-borne fungus that can cause the spoilage of many foods [3].

Most of the IDTs are potent tremor mammalian mycotoxins [4], that also exhibit excellent biological activities, including cytotoxic, antibacterial, antiviral, and protein tyrosine phosphatase inhibitory activities [5]. To date, some IDT compounds have been used for drug discovery. For example, as BK channel blockers, IDTs have been shown to reduce intraocular pressure and have been used to treat glaucoma [6]. The H1N1 virus is an invasive strain of influenza virus that can cause death in humans, and many IDTs have also shown significant activity against the H1N1 virus, especially emndole SB [7]. In agricultural settings, there has been a general trend toward using tremor-free IDTs as pesticides, such as 20,25-dihydroxyaflavinine [8].

According to previous reports, 3-GGI is a common precursor compound of all IDTs. The origin of the indole ring in its structure was clarified in 1983, when Jesus et al. studied the biosynthesis of penitrem A, the results of isotope labeling experiments showed that the indole part was derived from the IGP precursor of tryptophan[9]. In 2013, Tagami et al. analyzed a pentenyltransfer PaxC in paxilline biosynthetic gene cluster, and proved that indole ring derived from IGP through in vitro enzyme activity experiment [10]. Next, on this basis, IDT can be further divided into two types, namely the paspaline type with a large proportion and a small part of the non-paspaline type. There are some reviews related to IDTs have been presented. For example, the synthesis and activity of paspaline-type compounds [11, 12], the structural diversity and biological activity [5], biosynthesis of IDTs are described [13–15]. However, considering that these reviews do not classify these two types of compounds uniformly, and exclude the cover of the latest IDT compounds in recent years. So here, we renamed the IDT skeleton rings **A**, **B**, **C**, **D**, **E**, and **F**, and 77 non-paspaline skeleton and 127 paspaline skeleton IDTs were uniformly reclassified and summarized according to their structures and oxidative modifications. This review will contribute to the scientific community's comprehensive and compact understanding of the complex and diverse IDTs.

## Non-paspaline skeleton type

### Nodulisporic acid series

**Fig. 1:**
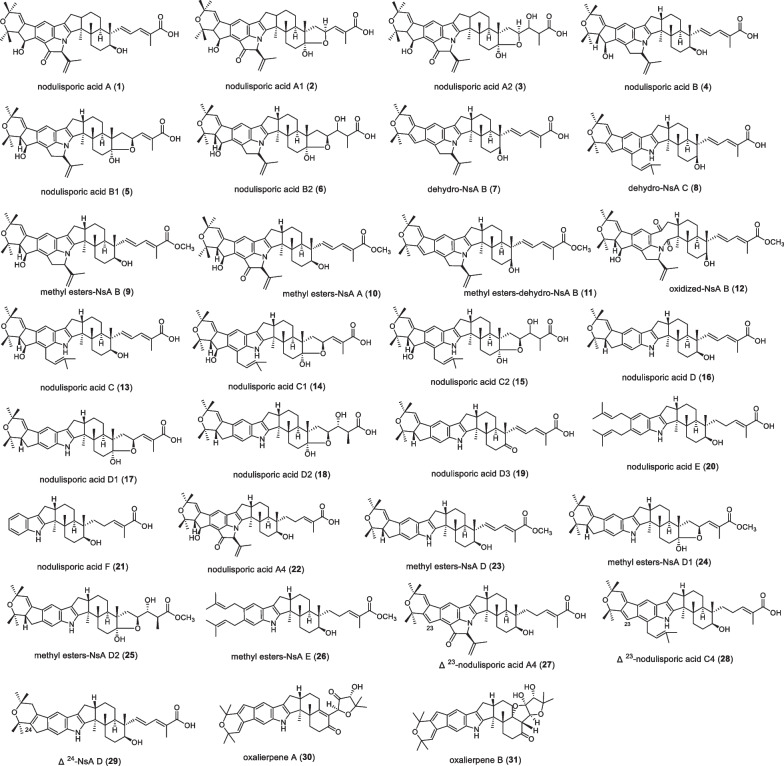
Chemical structures of nodulisporic acid series

A significant feature of this series is a caproic acid attached to the **E** ring, which contains 31 kinds of IDT compounds (Fig. 1 and Table 1). Nodulisporic acid A (NsA A, **1**) was discovered in *Hypoxylon* *pulicicidum* in 1992, and was first reported as a potent insecticide in 1997 [16]. It exhibits optimal activity with an LD90 (lethal dose 90%) of 1.5 μM in the flea assay and an IC_50_ (half maximal inhibitory concentration) of 0.00027 μM in the binding assay [17]. In 1999, Otto D et al. found the compounds nodulisporic acid A1 (NsA A1, **2**) and nodulisporic acid A2 (NsA A2, **3**) from *Nodulisporium* spp*.*, the LD50 (lethal dose 50%) of **2** to green flies was 0.3–1 μg/mL, like compound **1**. In the mosquito larvae assay, compound **2** was the strongest with an LD90 of 200 ng/mL [18], while **3** was slightly less active, with an LD50 of 0.6–1.5 μg/mL [19]. From *Nodulisporium* spp*.*, nodulisporic acid B (NsA B, **4**), nodulisporic acid B1 (NsA B1, **5**), nodulisporic acid B2 (NsA B2, **6**), dehydro-NsA B (**7**), dehydro-NsA C (**8**) and derivative-compound **9–12** were found in 2002, in which **4** was 100 fold less active on fleas than **1**. **5** is slightly more active than **6**. It was also found that while the methyl ester derivative of **11** was tenfold less active than the corresponding acid **1**, the activity of **5** and its methyl ester **10** was similar. **10** might be slightly more potent than **4**. However, compounds **5**, **6**, and **12** were inactive at 100 ppm (parts per million) [20]. In 2003, nodulisporic acid C (NsA C, **13**), nodulisporic acid C1 (NsA C1, **14**), nodulisporic acid C2 (NsA C2, **15**) were found. **13** showed good activity against fleas, which was 10 times lower than **1**, and the LD90 was 10 μg/ml; but compounds **14** and **15** had no activity in the flea test. The activity of compound **13** was significantly lower in mosquito larvae and fly maggot larvae assays (LD90 = 10,000 ng/mL) [21]. In 2004, nodulisporic acid D (NsA D, **16**), D1 (NsA D1, **17**), D2 (NsA D2, **18**), D3 (NsA D3, **19**), E (NsA E, **20**), F (NsA F, **21**), A4 (NsA A4, **22**) and compound **23–29** were successively discovered in the mutant strain *Nodulisporium* spp*.* In the flea assay test, compounds **16**, **20**, and **21** were 62, 12, and 30 fold less active than **1**, respectively. Nodulisporic acid containing a dienoic acid chain showed better activity in its series. For example, **1** is more active than **2** and **3**. However, in the NsA D series, the biological activity of **18** is significantly better than that of **16** and **17**. No biological activity was detected for compounds **23–29**, and*Δ*^23 or 24^-nodulisporic acids (**27**, **28**, **29**) were less active than corresponding nodulisporic acids of the same class [18]. In 2022, Zhang YH et al. isolated two specific compounds, oxalerpene A and B (**30** and **31)**, from *Penicillium* *oxalicum*. **30** is the first IDT derivative with a 4-hydroxy-5,5-dimethylhydrofuran-3-one in the five-membered side chain. **31** has a unique 6/5/6/6/6/6/6/5/5/5 ring system. Oxalerpene A and B have antiviral activity against H1N1 and respiratory syncytial virus (RSV) with IC_50_ values from 2.8 to 9.4 µM [22].

**Table 1:** Name, bioactivities and source of nodulisporic acid series

**Number**	Compound name	Biological activity	Species origin	References
**1**	Nodulisporic acid A	Insecticidal activity	*H.* *pulicicidum*	[16]
**2**	Nodulisporic acid A1	Insecticidal activity	*Nodulisporium* spp.	[19]
**3**	Nodulisporic acid A2	Insecticidal activity	*Nodulisporium* spp.	[19]
**4**	Nodulisporic acid B	Insecticidal activity	*Nodulisporium* spp.	[20]
**5**	Nodulisporic acid B1	Insecticidal activity	*Nodulisporium* spp.	[20]
**6**	Nodulisporic acid B2	Insecticidal activity	*Nodulisporium* spp.	[20]
**7**	Dehydro-NsA B		*Nodulisporium* spp.	[17]
**8**	Dehydro-NsA C		*Nodulisporium* spp.	[17]
**9**	Methyl esters-NsA B	Insecticidal activity	*Nodulisporium* spp.	[20]
**10**	Methyl esters-NsA A	Insecticidal activity	*Nodulisporium* spp.	[20]
**11**	Methyl esters-dehydro-NsA B	Less active	*Nodulisporium* spp.	[20]
**12**	Oxidized-NsA B	Insecticidal activity	*Nodulisporium* spp.	[20]
**13**	Nodulisporic acid C	Flea agent	*Nodulisporium* spp.	[21]
**14**	Nodulisporic acid C1		*Nodulisporium* spp.	[21]
**15**	Nodulisporic acid C2		*Nodulisporium* spp.	[21]
**16**	Nodulisporic acid D	Flea agent	*Nodulisporium* spp.	[18]
**17**	Nodulisporic acid D1	Flea agent	*Nodulisporium* spp.	[18]
**18**	Nodulisporic acid D2	Flea agent	*Nodulisporium* spp.	[18]
**19**	Nodulisporic acid D3		*Nodulisporium* spp.	[18]
**20**	Nodulisporic acid E	Insecticidal activity	*Nodulisporium* spp.	[18]
**21**	Nodulisporic acid F	Flea agent	*Nodulisporium* spp.	[18]
**22**	Nodulisporic acid A4	Insecticidal activity	*Nodulisporium* spp.	[18]
**23**	Methyl esters-NsA D		*Nodulisporium* spp.	[18]
**24**	Methyl esters-NsA D1		*Nodulisporium* spp.	[18]
**25**	Methyl esters-NsA D2		*Nodulisporium* spp.	[18]
**26**	Methyl esters-NsA E		*Nodulisporium* spp.	[18]
**27**	Δ^23^-NsA A4	Less active	*Nodulisporium* spp*.*	[18]
**28**	Δ^23^-NsA C4	Less active	*Nodulisporium* spp*.*	[18]
**29**	Δ^24^-NsA D		*Nodulisporium* spp*.*	[18]
**30**	Oxalierpene A	Antiviral	*P.oxalicum*	[22]
**31**	Oxalierpene B	Antiviral	*P.oxalicum*	[22]

### Emindole SB series

**Fig. 2:**
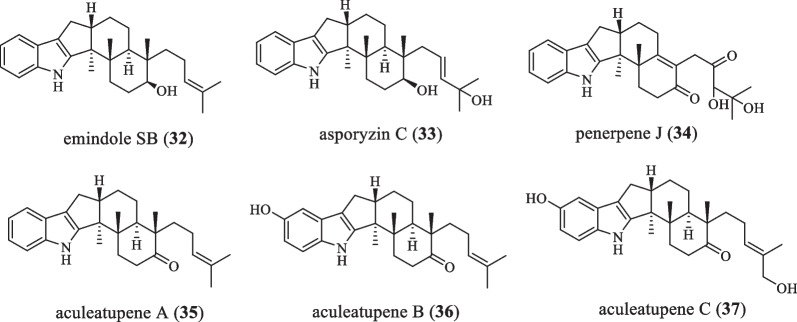
Chemical structures of emindole SB series

The emindole SB series is different from the Nodulisporic acid series in that there is no caproic acid on the E ring (Fig. 2 and Table 2). In 1966, the compound emindole SB (**32**) was isolated from *Claviceps* *paspali*, which was cytotoxic to cancer cell lines, and also showed antibacterial activity against *Staphylococcus* *aureus* ATCC 6538 and *Bacillus* *subtilis* ATCC 6633 [23–25]. In 2010, asporyzin C (**33**) was isolated from *Aspergillus* *oryzae*, and the antibacterial activity against *E.* *coli* as well as the antifungal activity against plant pathogens *Colletotrichum* *lagenarium* and *Fusarium* *oxysporium* were assayed. **33** exhibited intense activity against *E.* *coli* with an inhibitory diameter of 8.3 mm [25]. In 2020, the natural product penerpene J (**34**) was found in the fungus *Penicillium* sp. KFD28. This compound has inhibitory activity against both PTP1B (protein tyrosine phosphatase 1B) and TCPTP (protein tyrosine phosphatase), with IC_50_ values of 9.5 μM and 14.7 μM, respectively [26]. In 2021, Chaiyosang B et al. isolated three novel IDTs aculeatupenes A-C (**35**–**37**) from the mycelium of *Aspergillus* *aculeatus* KKU-CT2. Compounds **35** and **36** showed weak cytotoxicity against HelaS3, KB, HepG2, MCF-7, and A549 cancer cell lines with IC_50_ values of 11.12–67.81 μM. **37** showed weak cytotoxicity against the HelaS3 cell line with an IC_50_ value of 17.48 μM, but no cytotoxicity against the vero cell line. Moreover, it was also found to exhibit weak antifungal activity against *Bacillus* *cereus* [27].

**Table 2:** Name, bioactivities and source of emindole SB series

**Number**	Compound name	Biological activity	Species origin	References
**32**	Emindole SB	Anti-cancer, antibacterial	*C.* *paspali*	[24]
**33**	Asporyzin C	Antibacterial	*A.* *oryzae*	[25]
**34**	Penerpene J	Anti-cancer	*Penicillium* sp.KFD28	[26]
**35**	Aculeatupene A	Anti-cancer	*A.* *aculeatus* KKU-CT2	[27]
**36**	Aculeatupene B	Anti-cancer	*A.* *aculeatus* KKU-CT2	[27]
**37**	Aculeatupene C	Anti-cancer	*A.* *aculeatus* KKU-CT2	[27]

### Emindole DA series

**Fig. 3:**
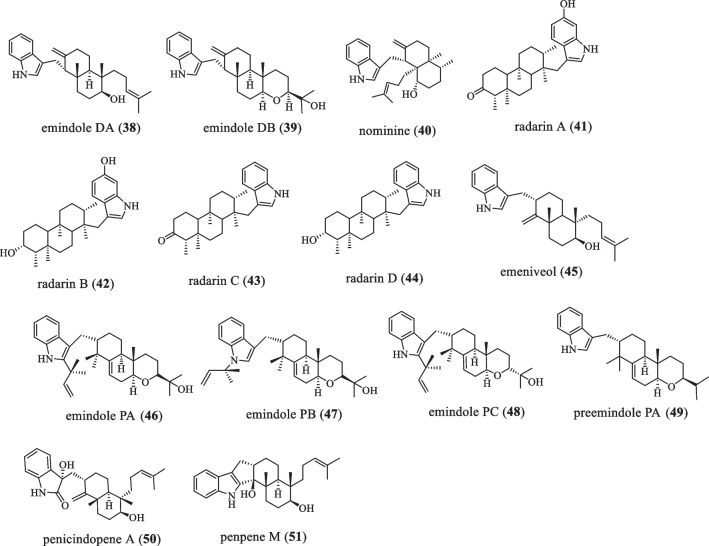
Chemical structures of emindole DA series

The common feature of this series of compounds is that they contain a 6/6-membered ring linked to a methylene group at the 3-position of the indole ring (Fig. 3 and Table 3). In 1988, the X-ray molecular structures of emindole DA (**38**) and DB (**39**) from *Emericella* *desertorum* were reported, both of which are tremor toxic to mammals [28, 29]. In 1989, nominine (**40**) was isolated as the leading organic soluble component of the sclerotium of the fungus *Aspergillus* *nomius* NRRL 13,137, which showed potent activity against the widespread crop pest *Heliothis* *zea*. When added to the standard test diet at 100 ppm dry weight, it resulted in 40% mortality and 97% weight loss relative to controls [30]. In 1992, compounds radarin A–D (**41**–**44**) were isolated from the fungus *Aspergillus* *sulphureus*. When added to a standard test diet of the corn worm *Helicoverpa* *zea* at 100 ppm, **41** reduced body weight gain by 52.7% relative to the control after 1 week. **43** also showed some activity at the same concentration, resulting in a 17.1% reduction in body weight gain. While **42** and **44** were inactive. Further biological evaluations were then performed to show that **41** was active against human lung cancer A549, breast cancer MCF7, and colon adenocarcinoma HT-29 cells with ED_50_ values of 2.5, 5.5, and 1.9 µg/mL, respectively. **42** was active in all three cell lines with ED_50_ (median effective dose) values of 2.0, 2.0,and 0.7 µg/mL, respectively [31]. In 1992, emeniveol (**45**) was isolated from *Emericella* *nivea*, and when the concentration was 100 mg/L, it could inhibit the germination of pine pollen and the growth of camellia pollen by about 35.5% [32]. In 2006, three IDTs were isolated from the mycelium of *Emericella* *purpurea*, namely emindoles PA (**46**), PB (**47**), and PC (**48**), among which **47** has strong anti-cancer activity [33]. Later, it found that its precursor compound preemindole PA (**49**) [13]. Liu L et al. isolated the compound penicindopene A (**50**) from *Penicillium* sp. YPCMAC1 in 2019, that showed moderate cytotoxicity to A549 and HeLa cell lines, with IC_50_ values of 15.2 and 20.5 µM, respectively [34]. In 2021, the compound penerpenes M (**51**) was discovered from the fungus *Penicillium* sp. KFD28. However, no antibacterial activity was found [35].

**Table 3:** Name, bioactivities and source of emindole DA series

**Number**	Compound name	Biological activity	Species origin	References
**38**	Emindole DA	Tremor toxin	*E.* *desertorum*	[28]
**39**	Emindole DB	Tremor toxin	*E.* *desertorum*	[28]
**40**	Nominine	Insect resistance	*A.* *nomius*	[30]
**41**	Radarin A	Insect resistance	*A.* *sulphureus*	[31]
**42**	Radarin B	Insect resistance	*A.* *sulphureus*	[31]
**43**	Radarin C	Insect resistance	*A.* *sulphureus*	[31]
**44**	Radarin D	Insect resistance	*A.* *sulphureus*	[31]
**45**	Emeniveol	Pollen growth inhibitor	*E.* *nivea*	[32]
**46**	Emindole PA		*E.* *purpurea*	[33]
**47**	Emindole PB	Anti-cancer	*E.* *purpurea*	[33]
**48**	Emindole PC		*E.* *purpurea*	[33]
**49**	Preemindole PA			[13]
**50**	Penicindopene A	cytotoxicity	*Penicillium* sp.	[34]
**51**	Penerpenes M		*Penicillium* sp.	[35]

### Aflavinine series

**Fig. 4:**
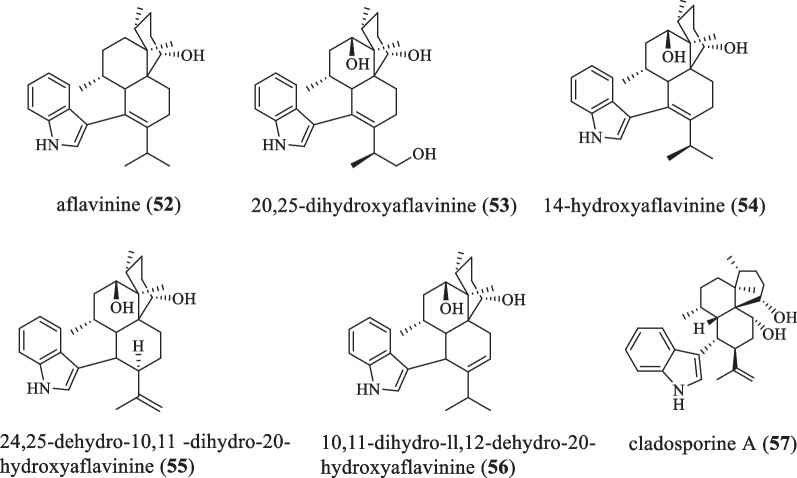
Chemical structures of aflavinine series

This series difference from the emindole DA series is that the 3-position of the indole ring is directly connected with the 6/6-membered ring (Fig. 4 and Table 4). In 1988, aflavinine (**52**) and its natural derivative products 20,25-dihydroxyaflavinine (**53**), 14-hydroxyflavinine (**54**), 24,25-dihydro-10,11-dihydro-20-hydroxyflavinine (**55**) and 10,11-dihydro-11,12-dihydro-20-hydroxyflavinine (**56**) were isolated from the fungus *Aspergillus* *flavus*, and these metabolites were selectively distributed to the sclerotia, It also showed antifeedant activity to fungus eating insects that usually encounter sclerotia in nature [8]. Compound **52** was non-toxic and non-tremor to 1-day-old chickens at 300 mg/kg. Compounds **54**–**56** were inactive against *C.* *hemipterus* at 100 ppm, but showed significant feeding deterrence when tested at the levels found in the sclerotia (400–1100 ppm). Compounds **53** and **54**–**56** also showed mild activity against *Bacillus* *subtilis* in a standard disk assay of 100 mg/disk, but were not toxic to brine shrimp at 250 mg/ml [36]. In 2019, Han X et al. isolated a new IDT cladosporine A (**57**) from the extract of the fungal strain *Cladosporium* sp*.* JNU17DTH12-9-01, which was the first report of the existence of IDT in *Cladosporium* spp. The MIC(minimum inhibitory concentration) of this compound to *Staphylococcus* *aureus* 209P and *Candida* *albicans* FIM 709 was 4 μg/mL and 16 μg/mL, respectively [37].

**Table 4:** Name, bioactivities and source of aflavinine series

**Number**	Compound name	Biological activity	Species origin	References
**52**	Aflavinine	Insect resistance	*A.* *tubingensis*	[36]
**53**	20,25-Dihydroxyaflavinine	Insect resistance	*A.* *flavus*	[8]
**54**	14-Hydroxyaflavinine	Insect resistance	*A.* *flavus*	[8]
**55**	24,25-Dehydro-10,11-dihydro-20-hydroxyaflavinine	Insect resistance	*A.* *flavus*	[8]
**56**	10,ll-Dihydro-ll,12-dehydro-20-hydroxyaflavinine	Insect resistance	*A.* *flavus*	[8]
**57**	Cladosporine A	Antibacterial	*Cladosporium* sp.	[37]

### Tubingensin A series

**Fig. 5:**
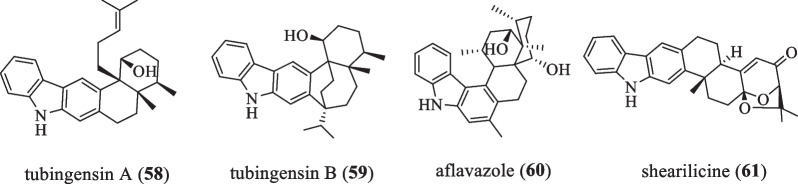
Chemical structures of tubingensin A series

The structure of this series is characterized by the presence of a benzene ring attached to the indole ring B (Fig. 5 and Table 5). In 1989, tubingensin A (**58**) and its structural isomer tubingensin B (**59**) were isolated from the fungus *Aspergillus* *tubingensis* by Gloer JB and colleagues, and **58** was found to be resistant to the general crop pest *Heliothis* *zea*, and exhibit showed in vitro antiviral activity against herpesvirus type I [38], while **59** showed mild activity against the crop pest *H.* *zea*, resulting in a 10% mortality rate when added to a standard diet at 125 ppm. The compound also showed almost identical activity to **58** in assays against herpes simplex virus type I with an IC_50_ of 9 μg/mL, but was more cytotoxic to HeLa cells (IC_50_ 4 μg/mL) [39]. In 1990, the compound aflavazole (**60**) was isolated from *Aspergillus* *flavus*. When added at 100 ppm to the standard test diet, **60** showed significant feeding-rejecting activity against the fungus-eating beetle *Carpophilus* *hemipterus* and was second only to dihydroxyaflavinine in activity against *C.* *hemipterus* among the IDT mycorrhizal metabolites of *A.* *flavus* [40]. When added to diets at concentrations found in *A.* *flavus* sclerotia (200–600 ppm), almost complete feeding deterrence was observed [40, 41]. In 2019, Miles CO and his colleagues isolated the compound shearilicine (**61**) from the strain *Penicillium* sp. ZO-R1-1, which had an IC_50_ value of less than 10 μM against L5178Y or A2780 cells, was tested against the human embryonic kidney cell line HEK-293. The results showed the highest selectivity in tests with SI (selectivity index) values in the range 3.3–8.1 and were also the most active metabolite against L5178Y cells with an IC_50_ value of 3.6 μM and A2780 cells with an IC_50_ value of 8.7 μM [42].

**Table 5:** Name, bioactivities and source of tubingensin A series

**Number**	Compound name	Biological activity	Species origin	References
**58**	Tubingensin A	Anti-insect, anti-virus	*A.* *tubingensis*	[38]
**59**	Tubingensin B	Cytotoxicity	*A.* *tubingensis*	[39]
**60**	Aflavazole	Anti-insect	*A.* *flavus*	[40]
**61**	Shearilicine	Cytotoxicity	*Penicillium* sp*.*	[42]

### Other non-paspaline skeleton type compounds

**Fig. 6:**
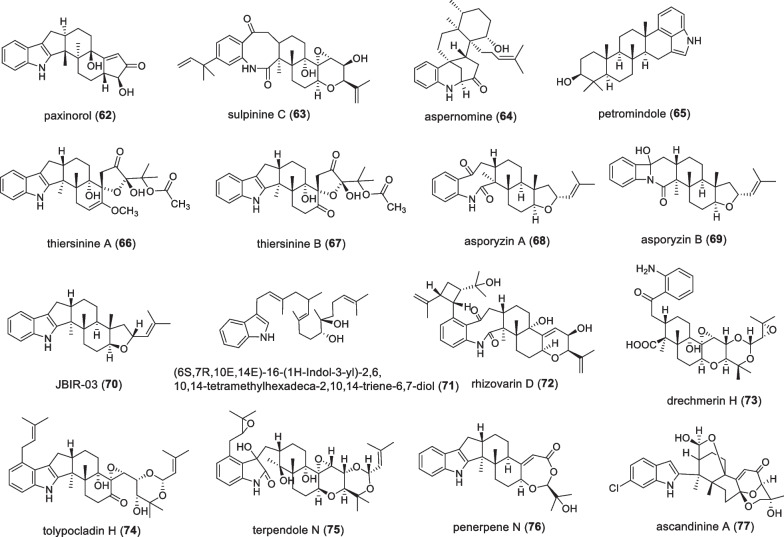
Chemical structures of other types of compounds

This series contain irregular non-paspaline type compounds (Fig. 6 and Table 6). In 1992, the compound paxinorol (**62**), isolated from the fungus *Penicillium* *paxilli*, which was found to be toxic to mammals, and it reduced the activity behavior of mice, but returned to normal after some time [43]. In the same year, the compound sulpinine C (**63**) was isolated from *Aspergillus* *sulphureus*, which was weakly active against *H.* *zea* but inactive against *C.* *hemipterus* [44]. In 1992, Gloer JB and his colleagues reported the anti-insect metabolite aspernomine (**64**) from *Aspergillus* *nomius*, which showed moderate activity against *H.* *zea*. Incorporating this compound at 100 ppm into the standard test diet resulted in a 35% reduction in body weight gain of the test insects relative to the control. Moreover, it also exhibited cytotoxicity against three human tumor cell lines, with ED_50_ values of 3.09, 4.93, and 3.08 μg/mL against A-549 lung, MCF-7 breast,and HT-29 colon adenocarcinoma cell lines, respectively [45]. In 1997, petromindole (**65**) was isolated by Ooike M et al. from the soil fungus *Petromyces* [46]. In 2002, two anthelmintic IDTs, thiersinine A (**66**) and B (**67**), were isolated from an organic extract of *P.* *thiersii* NRRL 28,147, which showed effective activity against *S.* *frugiperda* when added to standard test grains at 100 ppm, with growth compared to the control rates were reduced by 83% and 84% respectively. However, they were inactive against both *Candida* *albicans* ATCC 90,029 and *Staphylococcus* *aureus* ATCC 29,213 in the standard assay at 200 μg/plate [47]. In 2010, the natural products asporyzin A (**68**) and B (**69**) were isolated from *Aspergillus* *oryzae*, where **68** and **69** had lower insecticidal activity than their precursor JBIR-03, and neither of them showed any antifungal activity [25]. In 2010, the IDT JBIR-03 (**70**) was isolated from the fungus *Dichotomyces* *cejpii* *var*., which showed anti-MRSA (methicillin-resistant *Staphylococcus* *aureus*) activity and was tested at 32 and 64 mg/ml, respectively. Inhibits the growth of gram-positive and gram-negative bacteria at a concentration of any cytotoxic activity [48]. In 2013, the compound (6S,7R,10E,14E)-16-(1H-indol-3-yl)-2,6,10,14-tetramethylhexadeca-2,10,14-triene-6,7-diol (**71**) was isolated from an acid fungal strain *Penicillium* *camemberti* OUCMDZ-1492, which showed significant protection against H1N1 virus-induced cytopathic with IC_50_ values of 34.1 μM, respectively [7]. In 2016, Gao SS et al. discovered the compound rhizovarin D (**72**) from Rhizomucor *Mucor* *irregularis* QEN-189, which represents the most complex member of IDT derivatives [49]. In 2018, Zhao JC et al. isolated a new 1(2), 2(18)-diseco IDT drechmerin H (**73**) from the fermentation broth of *Drechmeria* sp*.* This compound exhibits a significant agonistic effect on the pregnane X receptor (PXR) with an EC_50_ (concentration for 50% of maximal effect) value of 134.91 ± 2.01 nM [50]. In 2019, the IDT tolypocladin H (**74**) was isolated from the strain *Tolypocladium* sp*.* XL115, the compound is active against the fungus *A.* *fragariae* with MIC values of 6.25–50 μg/mL; also active against all bacteria tested, the MIC value is 12.5–25 μg/mL, but no cytotoxicity [51]. In 2020, Nur EAA et al. isolated a new IDT terpendole N (**75**) from *Volutella* *citrinella* BF-0440, but no physiological activity was found [52]. In 2021, the compound penerpene N (**76**) was identified from the fungus *Penicillium* sp*.* KFD28, which represents a second paxilline-type IDT with a 1,3-dioxane ring, has a low cytotoxic effect on Hela cancer cell lines, and no antimicrobial activity was found [35]. In 2021, the compound ascandinine A (**77**) was isolated from the Antarctic sponge-derived fungus *Aspergillus* *candidus* HDN15-152, which has an unprecedented 2-oxabicyclo [2.2.2]octan-3-ol motif embedded in a pentacyclic system [53].

**Table 6:** Name, bioactivities and source of other types of compounds

Number	Compound name	Biological activity	Species origin	References
**62**	Paxinorol	Animal toxicity	*P.* *paxilli*	[43]
**63**	Sulpinine C	Anti-insect	*Aspergillus* *sulphureus*	[44]
**64**	Aspernomine	Anti-insect, anti-cancer	*A.* *Nomius*	[45]
**65**	Petromindole		*P.* *muricatus*	[46]
**66**	Thiersinine A	Anti-insect	*P.* *thiersii*	[47]
**67**	Thiersinine B	Anti-insect	*P.* *thiersii*	[47]
**68**	Asporyzin A	Insecticide	*A.* *oryzae*	[25]
**69**	Asporyzin B	Insecticide	*A.* *oryzae*	[25]
**70**	JBIR-03	Anti-MRSA	*D.* *cejpii*	[48]
**71**	(6S,7R,10E,14E)-16-(1H-Indol-3-yl)-2,6,10,14-tetramethylhexadeca-2,10,14-triene-6,7-diol	Cytotoxicity	*P.* *camemberti*	[7]
**72**	Rhizovarin D		*Mucor* *irregularis*	[49]
**73**	Drechmerin H	Agonistic effect on PXR	*Drechmeria* sp.	[50]
**74**	Tolypocladin H	Antibacterial activity	*Tolypocladium* sp. XL115	[51]
**75**	Terpendole N		*V.* *citrinella* BF-0440	[52]
**76**	Penerpene N	Anti-cancer	*Penicillium* sp.	[35]
**77**	Ascandinine A		*A.candidus*	[53]

## Paspaline skeleton type

### Only paspaline skeleton

**Fig. 7:**
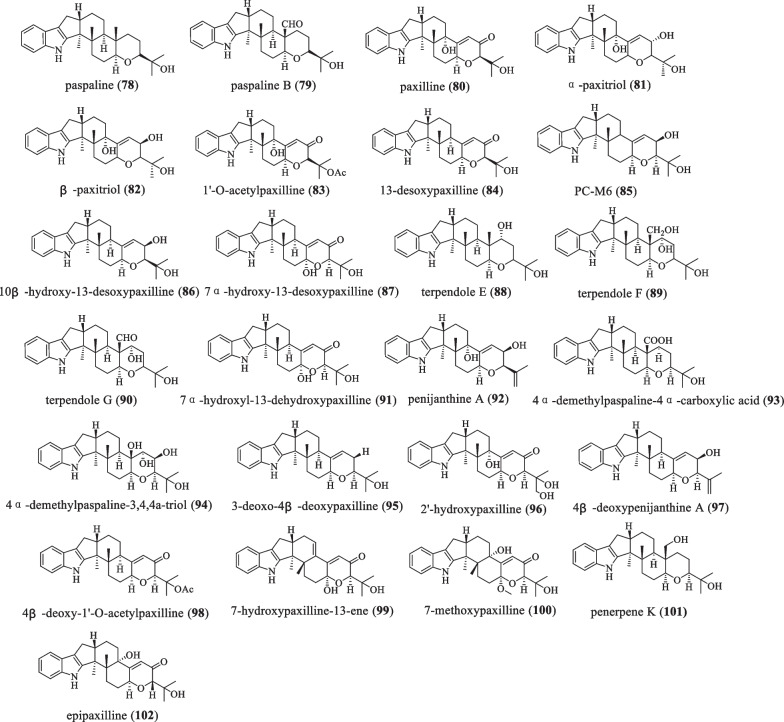
Chemical structures of paspaline-type compounds with only a paspaline skeleton

There are twenty-five IDT compounds containing only the paspaline skeleton (Fig. 7 and Table 7). In 1966, Arigoni D and his colleagues isolated the compound paspaline (**78**) from *Claviceps* *paspali*, which did not cause BK channel inhibition and tremor, but showed stronger anti-proliferative, anti-migratory, and Wnt/β-catenin inhibition than compound emindole SB [23, 54]. In the same year, Sarah et al. isolated the compound paspaline B (**79**) from the fungus *Penicilium* *paxilli*, which was the first oxidized analog of paspaline to be isolated, and also had tremor-causing activity for animals [55]. Paxilline (**80**) was first isolated from *P.* *paxilli* in 1974, and later Cole et al. reported that administration of 25 mg/kg of this compound caused severe intermittent tremors in roosters and mice [56, 57]. In 1989, Miles CO and colleagues discovered the compounds α-paxitriol (**81**) and β-paxitriol (**82**), neither of which caused tremors in mice [43]. In 1989, 1'-O-acetylpaxilline (**83**) was isolated from *Emericella* *striata*. When the injection concentration was 3.125 mg/kg, it could cause tremors in mice, and its tremor intensity was the same as that of paxilline. However, at the same time, it can also cause horn arch in mice [29]. In 1989, the compound 13-desoxypaxilline (**84**) was isolated from *Emericella* spp., which was active against human A-549 and HL-60 cancer cell lines, but had no antibacterial activity [35, 58]. In 1990, PC-M6 (**85**) was isolated from *P.* *crustosum*. The compound **85** showed moderate inhibitory activity against *Staphylococcus* *aureus* ATCC 6538, and also had activity for human gastric cancer cells [35, 59, 60]. In 1994, two compounds, 10β-hydroxy-13-desoxypaxilline (**86**) and 7α-hydroxy-13-desoxypaxilline (**87**), were isolated from the fungus *P.* *paxilli*, of which **86** showed significant resistance to human A-549 and HL-60 cancer cell lines, and it is the only paspaline-type IDTs that exhibits activity against both cell lines. **87** has tremor activity [61]. In 1995, Tomoda H et al. isolated and characterized terpendoles E (**88**), F (**89**), and G (**90**) from the culture broth of *Albophoma* *yamanashiensis* by using different production media [62]. They have a weak inhibitory effect on cholesterol acyltransferase (ACAT) activity, and **88** can be oxidatively modified to desoxyterpendole I (**123**) [63, 64]. In 1995, Belofsky et al. isolated the compound 7α-hydroxyl-13-dehydroxypaxilline (**91**) from *Eupenicillium* *Shearii* [65]*,* which showed moderate inhibitory activity against *Staphylococcus* *aureus* ATCC 6538 and antibacterial activity against *Bacillus* *subtilis* ATCC 6633 (MIC = 16 μg/mL), but showed no inhibitory activity against *E.* *coli* ATCC 25,922 and *L.* *monocytogenes* ATCC 1911 [35]. In 2009, the compound penijanthine A (**92**) was isolated from the fungus *Penicillium* *janthinellum*, which had no antifungal activity against *Aspergillus* *fumigatus* IFM 41,362, *Aspergillus* *niger* IFM 41,398, *Candida* *albicans* ATCC 90,028 or *Cryptococcus* *neoformans* ATCC 90,112 [66]. In 2013, 4α-demethylpaspaline-4α-carboxylic-acid (**93**) and 4α-demethylpaspaline-3,4,4α-triol (**94**) were isolated from an acid fungal strain *Penicillium* *camemberti* OUCMDZ-1492, and compound **94** was significant protection against H1N1 virus-induced cytopathic in MDCK cells with an IC_50_ value of 32.2 μM [7]. In 2013, the IDTs 3-deoxo-4β-deoxypaxilline (**95**) and 2'-hydroxypaxilline (**96**) were isolated from an acid fungal strain *Penicillium* *camemberti* OUCMDZ-1492, and compound **95** exhibited significant protection against H1N1 virus-induced cytopathic with IC_50_ value of 28.3 μM [7]. In 2014, the IDT 4β-deoxypenijanthine A (**97**) was isolated from the soil fungus *Penicillium* sp. CM-7, which showed no activity against human A-549 and HL-60 cancer cell lines [67]. In 2014, the IDT 4β-deoxy-1'-O-acetylpaxilline (**98**) was isolated from the soil fungus *Penicillium* sp. CM-7, which showed no effect on human A-549 and HL-60 cancer cell lines [67]. In 2019, during chemical research on the endophyte *Penicillium* sp. ZO-R1-1 was isolated from the medicinal plant ginger root, and the compounds 7-hydroxypaxilline-13-ene (**99**) and 7-methoxypaxilline (**100**) were discovered. Compound **99** showed cytotoxicity with IC_50_ values in the range of 5.3–8.1 μM [42]. In 2021, the IDT penerpene K (**101**) was isolated from a fermentation broth produced by adding L-tryptophan to the medium of the fungus *Penicillium* sp. KFD28. It has inhibitory activity against PTP1B and TCPTP, but has no antibacterial activity and cytotoxicity [35]. In 2021, the compound epi-paxilline (**102**) was isolated from the marine-derived fungus *Penicillium* sp*.*, which has inhibitory activity against PTP1B with IC_50_ values of 31.5 μM, respectively [26, 35].

**Table 7:** Name, bioactivities and source of compounds with only paspaline skeleton

Number	Compound name	Biological activity	Species origin	References
**78**	Paspaline	Anti-cancer	*C.* *paspali*	[23]
**79**	Paspaline B	Tremor activity	*Penicillium* sp.	[55]
**80**	Paxilline	Tremor and bacteriostatic activity	*P.* *paxilli*	[57]
**81**	α-Paxitriol	Tremor activity	*P.* *paxilli*	[43]
**82**	β-Paxitriol	Tremor activity	*P.* *paxilli*	[43]
**83**	1'-O-Acetylpaxilline	Tremor activity	*E.* *striata*	[29]
**84**	13-Desoxypaxilline	Anti-cancer	*Emericella* spp.	[58]
**85**	PC-M6	Antibacterial activity	*Penicillium* sp.	[59]
**86**	10β-Hydroxy-13-desoxypaxilline	Cell activity	*P.* *paxilli*	[61]
**87**	7α-Hydroxy-13-desoxypaxilline	Tremor activity	*P.* *paxilli*	[61]
**88**	Terpendole E	Mitotic kinesin	*Chaunopycnis* *alba*	[63]
**89**	Terpendole F	Weak activity	*A.* *yamanashiensis*	[62]
**90**	Terpendole G	Weak activity	*A.* *yamanashiensis*	[62]
**91**	7α-Hydroxyl-13-dehydroxypaxilline	Antibacterial activity	*E.* *Shearii*	[65]
**92**	Penijanthine A		*P.* *janthinellum*	[66]
**93**	4α-Demethylpaspaline-4α-carboxylic acid	Cytotoxicity	*P.* *camemberti*	[7]
**94**	4α-Demethylpaspaline-3,4,4α-triol	Cytotoxicity	*P.* *camemberti*	[7]
**95**	3-Deoxo-4β-deoxypaxilline	Cytotoxicity	*camemberti*	[7]
**96**	2′-Hydroxypaxilline		*P.* *camemberti*	[7]
**97**	4β-Deoxypenijanthine A		*Penicillium* sp.	[67]
**98**	4β-Deoxy-1′-O-acetylpaxilline		*Penicillium* sp.	[67]
**99**	7-Hydroxypaxilline-13-ene	Cytotoxicity	*Penicillium* sp.	[42]
**100**	7-Methoxypaxilline		*Penicillium* sp.	[42]
**101**	Penerpene K	Inhibitory activity against PTP1B and TCPTP	*Penicillium* sp.	[35]
**102**	Epipaxilline	Anti-cancer	*Penicillium* sp.	[26]

### F-ring modification

**Fig. 8:**
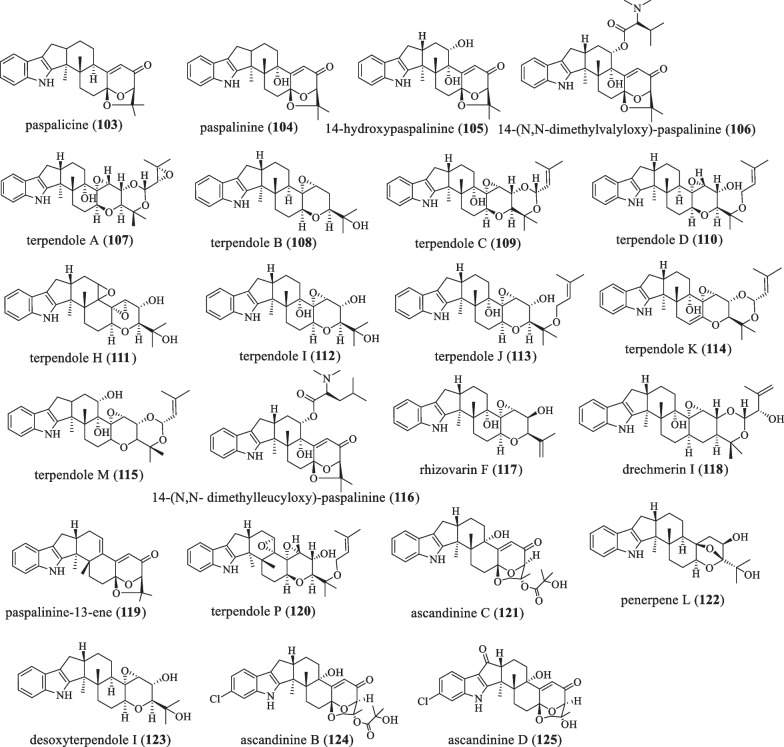
Chemical structures of paspaline-type compounds with F-ring modification

Based on the paspaline skeleton, its **F**-ring was modified by epoxidation (Fig. 8 and Table 8). In 1966, Arigoni D and his colleagues isolated the compound paspalicine (**103**) from *Claviceps* *paspali*, a dehydroxylated analog of paspalinine lacking tremor activity [23]. It can effectively block maxi-K (high-conductance Ca^2+^-activated K^+^) channels [24, 54]. In 1980, Gallagher RT et al. discovered the compound paspalinine (**104**) from *Claviceps* *paspali*, a mycotoxin that causes tremors in mice [68]. In 1993, compounds 14-hydroxypaspalinine (**105**) and 14-(N, N-dimethylvalyloxy)-paspalinine (**106**) were isolated from the fungus *Aspergillus* *nomius*. At 100 ppm levels, the two compounds resulted in a 90% reduction in body weight gain in tests against the corn roundworm *H.* *zea*. However, at this concentration, **105** does not have any effect [69]. In 1995, Huang XH et al. isolated and characterized terpendole A (**107**), B (**108**), C (**109**), and D (**110**) from *Albophoma* *yamanashiensis* and found that they showed strong inhibition of ACAT activity [70]. In 1995, Tomoda et al. isolated and characterized terpendoles H–K (**111**–**114**) from the culture broth of *A.* *yamanashiensis* using different production media [62]. **113** and **114** have moderate inhibitory effects on ACAT activity with IC_50_ values of 38.8 μm and 38.0 μm in rat liver microsomes, respectively, but **114** has a weaker activity [62–64]. In 1999, terpendole M (**115**) was isolated from perennial ryegrass (*Lolium* *perenne*) infected with the endophytic fungus *Neotyphodium* *lolii*. In standard mouse bioassays, this compound was less tremor than **109** [71]. In 2006, Junker et al. isolated and discovered the compound 14-(N,N-dimethylleucyloxy)-paspalinine (**116**) from *Aspergillus* *alliaceus* culture medium by optimizing the culture conditions [72]. In 2016, Gao et al. discovered the compound rhizovarins F (**117**) from Rhizomucor *Mucor* *irregularis* QEN-189 [49]. In 2019, Liang JH and colleagues isolated a new IDT drechmerin I (**118**) from the fermentation broth of *Drechmeria* sp*.*, which has antibacterial activity against *Bacillus* *subtilis* with a MIC value of 200 μg/mL [73]. In 2019, during chemical research on the endophyte *Penicillium* sp*.* ZO-R1-1 isolated from the root of the medicinal plant ginger, paspalinine-13-ene (**119**), was discovered, which shows cytotoxicity with IC_50_ values in the range of 5.3–8.1 μM [42]. In 2020, the compound terpendole P (**120**) was isolated from the culture medium of the fungus *Volutella* *citrinella* BF-0440, which has 6 consecutive ring systems and an indole ring and can inhibit sterol O-acyltransferases 1 and 2 (SOAT1 and 2) [52]. In 2021, the compound ascandinine C (**121**) was isolated from the Antarctic sponge-derived fungus *Aspergillus* *candidus* HDN15-152. It is a rare IDT with a 6/5/5/6/6/6/6-fused ring system. The compound **121** has anti-influenza virus A (H1N1) activity with an IC_50_ value of 26 μM [53]. In 2021, the IDT penerpene L (**122**) was isolated from a fermentation broth produced by adding L-tryptophan to the medium of the fungus *Penicillium* *sp.* KFD28. It has inhibitory activity against PTP1B and TCPTP, but has no antibacterial activity and cytotoxicity [35]. In the same year, the IDTs ascandinine B (**124**) and D (**125**) were isolated from the Antarctic sponge-derived fungus *Aspergillus* *candidus* HDN15-152. They represent a rare IDT with a 6/5/5/6/6/6/6-fused ring system. Among them, **125** is cytotoxic to HL-60 cells with an IC_50_ value of 7.8 μM [53].

**Table 8:** Name, bioactivities and source of compounds with F-ring modification

Number	Compound name	Biological activity	Species origin	References
**103**	Paspalicine	Block maxi-K channels	*C.* *paspali*	[23]
**104**	Paspalinine	Vibratory mycotoxins	*P.* *tularense*	[68]
**105**	14-Hydroxypaspalinine	Insect resistance	*Aspergillus* *nomius*	[69]
**106**	14-(N,N-dimethylvalyloxy)-Paspalinine	Insect resistance	*Aspergillus* *nomius*	[69]
**107**	Terpendole A	ACAT inhibitors	*A.* *yamanashiensis*	[70]
**108**	Terpendole B	ACAT inhibitors	*A.* *yamanashiensis*	[70]
**109**	Terpendole C	Tremor activity	*A.* *yamanashiensis*	[70]
**110**	Terpendole D	ACAT inhibitors	*A.* *yamanashiensis*	[70]
**111**	Terpendole H	Weak activity	*A.* *yamanashiensis*	[62]
**112**	Terpendole I	ACAT inhibitors	*A.* *yamanashiensis*	[62]
**113**	Terpendole J	ACAT inhibitors	*A.* *yamanashiensis*	[62]
**114**	Terpendole K	Tremor activity	*I.* *muelleri*	[62]
**115**	Terpendole M	ACAT inhibitors	*N.* *lolii*	[71]
**116**	14-(N,N-dimethylleucyloxy)-Paspalinine		*Aspergillus* *alliaceus*	[72]
**117**	Rhizovarin F		*Mucor* *irregularis*	[49]
**118**	Drechmerin I	Antibacterial activity	*Drechmeria* sp.	[73]
**119**	Paspalinine-13-ene	Cytotoxicity	*Penicillium* sp.	[42]
**120**	Terpendole P	Suppress SOAT	*Volutella* *citrinella*	[52]
**121**	Ascandinine C	Cytotoxicity	*A.* *candidus*	[53]
**122**	Penerpene L	Inhibitory activity against PTP1B and TCPTP	*Penicillium* sp.	[35]
**123**	Desoxyterpendole I			[13]
**124**	Ascandinine B		*A.* *candidus*	[53]
**125**	Ascandinine D	Cytotoxicity	*A.* *candidus*	[53]

### A-ring prenylation

**Fig. 9:**
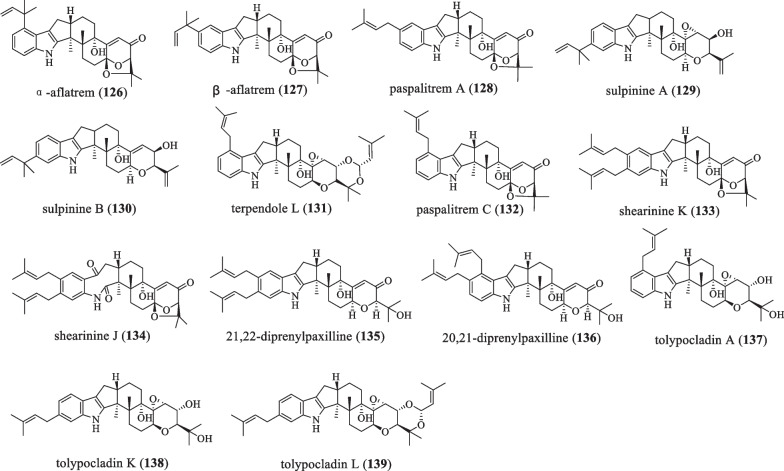
Chemical structures of paspaline-type compounds with A-ring prenylation

Based on the paspaline skeleton, the modification of isopentenyl was added to the 20, 21 or (and) 22 positions of the **A** ring of its indole ring (Fig. 9 and Table 9). In 1964, Wilson BJ isolated the compounds ɑ-aflatrem (**126**) and β-aflatrem (**127**) from *Aspergillus* *flavus*, which are fibrillating mycotoxins with acute neurotoxic effects [74, 75]. In 1977, Cole RJ et al. discovered the compound paspalitrem A (**128**) from *Claviceps* *paspali*, a toxin that can vibrate muscles [76, 77]. In 1992, compounds sulpinine A (**129**) and B (**130**) were isolated from *Aspergillus* *sulphureus*, both of which were active against *H.* *zea* but not against *C.* *hemipterus* [44]. Among them, **129** have the most potent activity. When this compound was added to the standard test diet at 100 ppm, a 96.0% reduction in body weight gain compared to the control was noted after one week, and a 10% mortality rate was also observed in this assay. **130** brought a similar weight gain reduction of 87.2%. Moreover, **129** was also cytotoxic to human lung cancer A549, breast cancer MCF7, and colon adenocarcinoma HT-29 cells with ED_50_ values of 25.7, 58.1, and 3.7 µg/ mL [44, 78]. In 1995, Tomoda H et al*.* isolated and characterized terpendole L (**131**) from the culture broth of *Albophoma* *yamanashiensis* by using different production media [62]. This compound has a moderate inhibitory effect on ACAT activity with an IC_50_ value of 32.4 μM in rat liver microsomes [62–64]. In 1996, the first systematic study of the effect of paspalitrem C (**132**) on the spontaneous contractile activity of a variety of mammalian smooth muscles [79], increased the spontaneous contractility of the bladder and duodenum in guinea pigs and rats, and caused tracheal tension in guinea pigs. These effects are attributed to blocking high conductance, Ca^2+^-activated K^+^ channels [77, 79]. In 2007, shearinine K (**133**) and J (**134**) were isolated and characterized from the endophytic fungus *Penicillium* sp*.* [80]. In 2013, the IDT 21,22-diprenylpaxilline (**135**) was isolated from an acid fungal strain *Penicillium* *camemberti* OUCMDZ-1492, which exhibits significant protection against H1N1 virus-induced cytopathic in MDCK cells with an IC_50_ value of 73.3 μM [7]. In 2014, when studying JanD and AmyD protein function, the compound 20,21-diprenylpaxilline (**136**) were discovered [13, 81, 82]. In 2019, the isoprene IDT tolypocladin A (**137**) was isolated from the fungus *Tolypocladium* sp*.*, which showed no inhibitory activity against three pathogenic fungi (*F.* *oxysporum*, *A.* *solani*, and *R.* *solani*). However, it showed significant inhibitory activity against seven pathogenic fungi (*A.* *fragariae,* *C.* *cassiicola,* *A.* *alternata,* *B.* *cinereal,* *C.* *personata,* *V.* *dahliae* *Kleb,* and *S.* *sclerotiorum*), with MIC values of 6.25–25 μg /mL. It is also active against *Bacillus* *cereus* and *Staphylococcus* *aureus*,with MIC values of 25 and 12.5 μg/mL, respectively [51]. In 2019, two new prenylated IDTs, namely tolypocladin K (**138**) and L (**139**), were isolated from the fungus *Tolypocladium* sp*.* XL115. The compound **138** exhibits moderate antifungal activity against *S.* *sclerotiorun,* *H.* *maydis,* *B.* *cinereal,* and *C.* *acutatum* with a MIC value of 50 μg/mL [64].

**Table 9:** Name, bioactivities and source of compounds with A-ring prenylation

Number	Compound name	Biological activity	Species origin	References
**126**	α-Aflatrem	Neurotoxicity	*A.* *flavus*	[74]
**127**	β-Aflatrem	Neurotoxicity	*A.* *flavus*	[74]
**128**	Aaspalitrem A	Tremor muscle toxin	*C.* *paspali*	[76]
**129**	Sulpinine A	Anti-insect, cytotoxic	*A.* *sulphureus*	[44]
**130**	Sulpinine B	Anti-insect	*A.* *sulphureus*	[44]
**131**	Terpendole L	Antibacterial activity	*Tolypocladium* sp*.*	[64]
**132**	Paspalitrem C	Tremor muscle toxin	*Phomopsis* sp.	[79]
**133**	Shearinine K		*P.* *janthinellum*	[80]
**134**	Shearinine J		*Penicillium* sp.	[80]
**135**	21,22-Diprenylpaxilline	Cytotoxic	*P.* *camemberti*	[7]
**136**	20,21-Diprenylpaxilline			[81]
**137**	Tolypocladin A	Antibacterial activity, cytotoxic	*Tolypocladium* sp*.*	[51]
**138**	Tolypocladin K	Antifungal activity	*Tolypocladium* sp.	[64]
**139**	Tolypocladin L		*Tolypocladium* sp.	[64]

### The isopentenyl group on the A ring is modified

**Fig. 10:**
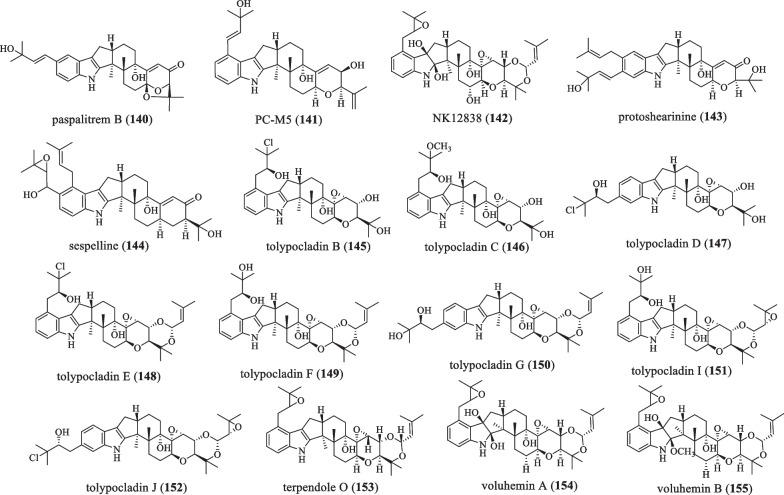
Chemical structures of paspaline-type compounds in which the isopentenyl group on the A-ring is modified

The difference from the previous classification is that the isopentenyl group on the **A** ring is further modified by oxidation, halogenation, or epoxidation (Fig. 10 and Table 10). In 1977, Cole et al. discovered the compound paspalitrem B (**140**) from *Claviceps* *paspali* [76]. Cattle are affected by tremors (also known as "staggering") as they graze on toxic pastures; the compound identified at the highest concentration was the compound **140** (~ 150 mg/kg) in *Claviceps* *cynodontis*-infected *Cynodon* *dactylon* collected from pastures causing staggered syndrome in South African cattle herds [76, 77]. In 1990, PC-M5 (**141**) was isolated from *Penicillium* *crustosum*, which is toxic to PC12 cells [35, 59, 60]. In 2002, Tsuchiya et al. found the isolated and characterized compound NK12838 (**142**), which inhibits the activities of SOAT1 and SOAT2 with a SI value (log (IC_50_ for SOAT1)/(IC_50_ for SOAT2)) of + 0.27, but has no cytotoxicity [83, 84]. In 2016, while studying the biosynthesis of shearinine, the compound protoshearinine (**143**) was characterized [85]. In 2018, the compound sespelline (**144**) was reported while studying the biosynthesis of sespendole [86]. In 2019, new isoprenindole diterpenes tolypocladins B–G (**145**–**150**), I (**151**), and J (**152**) were isolated from the fungus *Tolypocladium* sp*.*, they showed no inhibitory activity against three pathogenic fungi (*F.* *oxysporum*, *A.* *solani* and *R.* *solani*). All of them are active against *A.* *fragariae* with MIC values of 6.25–50 μg/mL, and compound **145** has weak activity against *Staphylococcus* *aureus* [51]. In 2020, the compound terpendole O (**153**) was isolated from the culture medium of the fungus *Volutella* *citrinella* BF-0440, which has 7 consecutive ring systems and an indole ring. It can inhibit sterol SOAT1 and 2 [52]. In 2020, Ohshiro T and colleagues isolated new compounds, termed voluhemins A (**154**) and B (**155**), from the culture broth of the fungal strain *Volutella* *citrinella* BF 0440. They have a common IDT core and two additional isoprenyl moieties, and **155** are O-methylated **154**. **154** can inhibit the activities of SOAT1 and SOAT2 with a SI value of + 0.45, and **155** can selectively inhibit the SOAT2 isoenzyme. However, none of which is cytotoxic [83].

**Table 10:** Name, bioactivities and source of compounds with the isopentenyl group on the A-ring is modified

Number	Compound name	Biological activity	Species origin	References
**140**	Paspalitrem B	Tremor muscle toxin	*C.* *paspali*	[76]
**141**	PC-M5	Cytotoxicity	*P.* *crustosum*	[59]
**142**	NK12838	Inhibits SOAT1 and SOAT2 activity	*V.* *citrinella*	[84]
**143**	Protoshearinine			[85]
**144**	Sespelline			[86]
**145**	Tolypocladin B	Antibacterial activity	*Tolypocladium* sp.	[51]
**146**	Tolypocladin C	Antibacterial activity	*Tolypocladium* sp.	[51]
**147**	Tolypocladin D	Antibacterial activity	*Tolypocladium* sp.	[51]
**148**	Tolypocladin E	Antibacterial activity	*Tolypocladium* sp.	[51]
**149**	Tolypocladin F	Antibacterial activity	*Tolypocladium* sp.	[51]
**150**	Tolypocladin G	Antibacterial activity	*Tolypocladium* sp.	[51]
**151**	Tolypocladin I	Antibacterial activity	*Tolypocladium* sp.	[51]
**152**	Tolypocladin J	Antibacterial activity	*Tolypocladium* sp.	[51]
**153**	Terpendole O	Suppress SOAT	*Volutella* *citrinella*	[52]
**154**	Voluhemin A	Inhibits SOAT1 and SOAT2 activity	*V.* *citrinella*	[83]
**155**	Voluhemin B	Inhibit SOAT2 activity	*V.* *citrinella*	[83]

### A-ring with 6/5 member ring

**Fig. 11:**
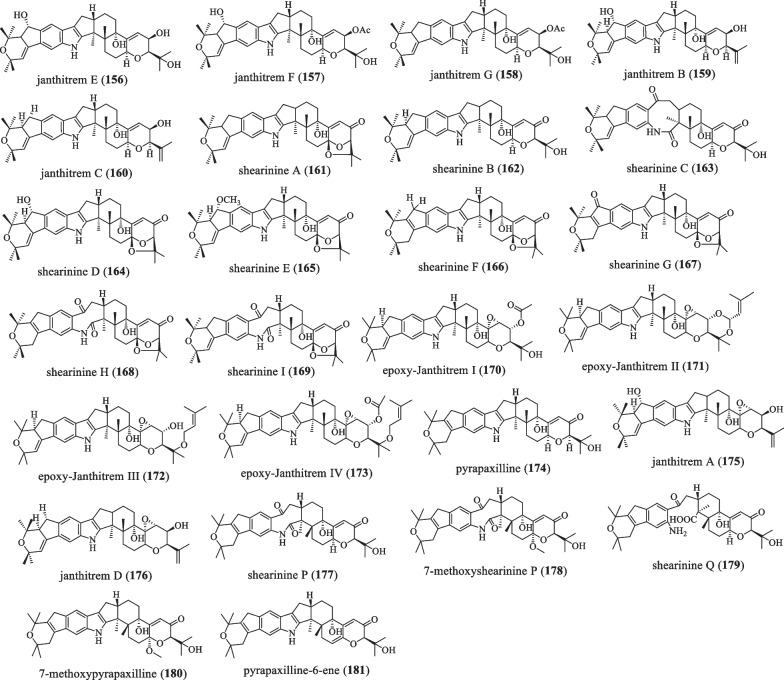
Chemical structures of paspaline-type compounds with A-ring 6/5 member ring

The 21 and 22 positions of the **A**-ring of the indole ring are modified with diprenyl groups, and then further oxidatively cyclized into a 6/5-membered ring (Fig. 11 and Table 11). In 1984, Jesus et al. isolated and identified the tremor toxin janthitrems E–G (**156**–**158**) from the fungus *P.* *janthinellum* [87]. In 1992, Wilkins et al. isolated janthitrem B (**159**) [88]. In 1993, Penn and colleagues isolated and identified the compound janthitrem C (**160**) [89]. In 1995, compounds shearinines A (**161**) and B (**162**) were discovered from the fungus *Eupenicillium* *shearii*, both of which showed potent activity against *H.* *zea* and *Carpophilus* *hemipterus* [65]. **161** also induces apoptosis in human leukemia HL-60 cells, while **162** causes significant mortality in leaf disc assays against *Spodoptera* *frugiperda* [65, 90]. In 1995, Belofsky et al. discovered the compound shearinine C (**163**) from *Eupenicillium* *Shearii*, which can be formed from **160** through an autoxidative process, which has anti-insect activity [65]. In 2007, Smetanina OF and colleagues isolated shearinines D(**164**), E(**165**),and F(**166**) from marine-derived strains of the fungus *Penicillium* *janthinellum* [90]. **166** inhibits EGF-induced malignant transformation of JB6 P^+^ Cl 41 cells in soft agar with INCC50 (inhibition of colony number 50) equal to 13 μM concentration. It may be a strongly effective cancer preventive agent in humans or animals. **164** and **165** induce apoptosis in human leukemia HL-60 cells at a concentration of 100 μM. Moreover, the apoptosis rates of apoptotic cells are 39% and 34%, respectively, compared with control cells [80, 90]. In 2007, shearinines D–G was isolated and characterized from the endophytic fungus *Penicillium* sp*.*, in which shearinine G (**167**) had inhibitory effects on BK channels [80]. Shearinines H (**168**) and I (**169**) were isolated and characterized from the endophytic fungus *Penicillium* sp*.* in 2007 [80]. In 2010, the compound epoxy-janthitrems I–IV (**170**–**173**) was isolated and identified from the endophyte *Epichloë* *endophytes*, and the compound epoxy-Janthitrems produced by the endophyte had strong inhibitory activity against insect larvae. However, when ryegrass plants are grown at a constant low temperatures for a long time, the concentration of the compounds in the plants is significantly reduced, and the insect resistance is less effective [91, 92]. In 2014, the compound pyrapaxilline (**174**) was isolated from *Eupenicillium* *shearii.* Lipopolysaccharide (LPS) increases NO production by approximately 2.5-fold over basal levels. When the mouse macrophage cell line RAW264.7 was pretreated with this compound for 2 h before LPS stimulation, it inhibited NO production by 40% at 10–30 μg/ml with no toxicity [93]. In 2018, new compounds 11,12-epoxyjanthitrem B and 11,12-epoxyjanthitrem C were isolated from the fungus *Penicillium* *janthinellum*, and named janthitrem A (**175**) and janthitrem D (**176**), respectively. Injecting mice with **175** at a concentration of 4 mg/kg can achieve high-intensity tremor effects in 15 min [94]. In 2019, Ariantari NP and colleagues isolated compounds shearinine P (**177**), 7-methoxyshearinine P (**178**), and shearinine Q (**179**) from strain *Penicillium* *sp.* ZO-R1-1. Among them, the IC_50_ value of **179** on L5178Y or A2780 cells is 10 μM [42]. In 2019, during chemical research on the endophyte *Penicillium* *sp.* ZO-R1-1 isolated from the medicinal plant ginger root, the compounds 7-methoxypyrapaxilline (**180**) and pyrapaxilline-6-ene (**181**) were discovered. Among them, the compound **181** showed cytotoxicity with IC_50_ values in the range of 5.3–8.1 μM; and also showed significant cytotoxic activity against the A2780 human ovarian cancer cell line, with IC_50_ values of 5.3–8.7 μM [42].

**Table 11:** Name, bioactivities and source of compounds with A-ring 6/5 member ring

Number	Compound name	Biological activity	Species origin	References
**156**	Janthitrem E	Tremor toxin	*P.* *janthinellum*	[87]
**157**	Janthitrem F	Tremor toxin	*P.* *janthinellum*	[87]
**158**	Janthitrem G	Tremor toxin	*P.* *janthinellum*	[87]
**159**	Janthitrem B	Tremor, anti-insect activity	*P.* *janthinellum*	[88]
**160**	Janthitrem C		*P.* *janthinellum*	[89]
**161**	Shearinine A	Anti-insect, cancer cell activity	*E.* *shearii*	[65]
**162**	Shearinine B	Anti-insect activity	*E.* *shearii*	[65]
**163**	Shearinine C	anti-insect activity	*E.* *shearii*	[65]
**164**	Shearinine D	cancer cell activity	*P.* *janthinellum*	[80]
**165**	Shearinine E	Anti-cancer	*P.* *janthinellum*	[80]
**166**	Shearinine F		*Penicillium* sp.	[80]
**167**	Shearinine G	BK channel inhibition	*Penicillium* sp.	[80]
**168**	Shearinine H		*Penicillium* sp.	[80]
**169**	Shearinine I		*Penicillium* sp.	[80]
**170**	Epoxy-Janthitrem I	Pesticide	*E.* *endophytes*	[91]
**171**	Epoxy-Janthitrem II	Pesticide	*E.* *endophytes*	[91]
**172**	Epoxy-Janthitrem III	Pesticide	*E.* *endophytes*	[91]
**173**	Epoxy-Janthitrem IV	Pesticide	*E.* *endophytes*	[91]
**174**	Pyrapaxilline	Inhibit the production of NO	*E.* *shearii*	[93]
**175**	Janthitrem A	Tremor, anti-insect activity	*P.* *janthinellum*	[94]
**176**	Janthitrem D		*P.* *janthinellum*	[94]
**177**	Shearinine P		*Penicillium* sp.	[42]
**178**	7-Methoxyshearinine P		*Penicillium* sp.	[42]
**179**	Shearinine Q		*Penicillium* sp.	[42]
**180**	7-Methoxypyrapaxilline		*Penicillium* sp.	[42]
**181**	Pyrapaxilline-6-ene	Cytotoxicity	*Penicillium* sp.	[42]

### A-ring with 5/6 member ring

**Fig. 12:**
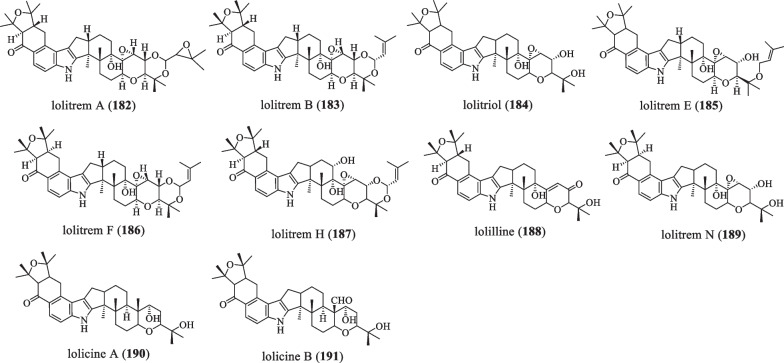
Chemical structures of paspaline-type compounds with A-ring 5/6 member ring

The difference from the previous type is that this type is further oxidatively cyclized into a 5/6-membered ring based on the diprenyl modification at the 20 and 21 positions of the **A** ring of the indole ring (Fig. 12 and Table 12). In 1981, two strong neurotoxins, lolitrems A (**182**) and B (**183**), were isolated from herbs that developed a livestock disease known as "ryegrass staggered disease." They can poison livestock with tremors that do not directly impair spatial learning and memory, but reduce voluntary movements in poisoned animals; later, perennial ryegrass toxicosis (PRGT) was prevented by limiting the concentration of **183** [95]. In 1992, lolitriol (**184**) was found in extracts of endophyte-infected ryegrass leaves and cultures of *A.* *lolii* [43]. Moreover, **183** is quickly degraded to compound **184**, which does not cause tremors even at 20 mg/kg, so its activity is at least 20-fold lower than **183** [96]. In 1994, Christopher et al. obtained the abundant secondary compound lolitrem E (**185**) when **183** was purified from ryegrass staggers (RGS), which has intense BK channel activity but no tremor effect in animals [97, 98]. In 1996, Sarah et al. isolated lolitrem F (**186**), a stereoisomer of the vibratory mycotoxin **183**, from ryegrass infected with *Acremonium* *Lolii*. The compound **186** was found to have similar potency and duration of action as **183** in standard mouse bioassays, but was slightly less active than **183** [99]. In 1997, the compound lolitrem H (**187**) was discovered [71]. In 1997, Sarah et al. isolated lolilline (**188**) from an extract of ryegrass seeds infected with the endophytic fungus *Acremonium* *lolii*, which does not have tremor effects [100]. In 1998, lolitrem N (**189**), lolicine A (**190**), and B (**191**) were identified in an extract of perennial ryegrass (*Lolium* *perenne*) seeds infected with the endophytic fungus *Neotyphodium* *lolii*, and they are lolitrem-like compounds [101].

**Table 12:** Name, bioactivities and source of compounds with A-ring 5/6 member ring

Number	Compound name	Biological activity	Species origin	References
**182**	Lolitrem A	Neurotoxin	*E.* *festucae*	[95]
**183**	Lolitrem B	Neurotoxin	*E.* *festucae*	[95]
**184**	Lolitriol	Neurotoxin	*Endophyte-infected* *ryegrass*	[43]
**185**	Lolitrem E	BK channel activity	*Endophyte-infected* *perennial* *ryegrass*	[97]
**186**	Lolitrem F	Neurotoxin	*L.* *perenne*	[99]
**187**	Lolitrem H		*A.* *lolii*	[71]
**188**	Lolilline		*A.* *lolii*	[100]
**189**	Lolitrem N		*Endophyte-infected* *ryegrass*	[101]
**190**	Lolicine A		*L.* *perenne*	[101]
**191**	Lolicine B		*L.* *perenne*	[101]

### A-ring 4/5 or 6 membered ring

**Fig. 13:**
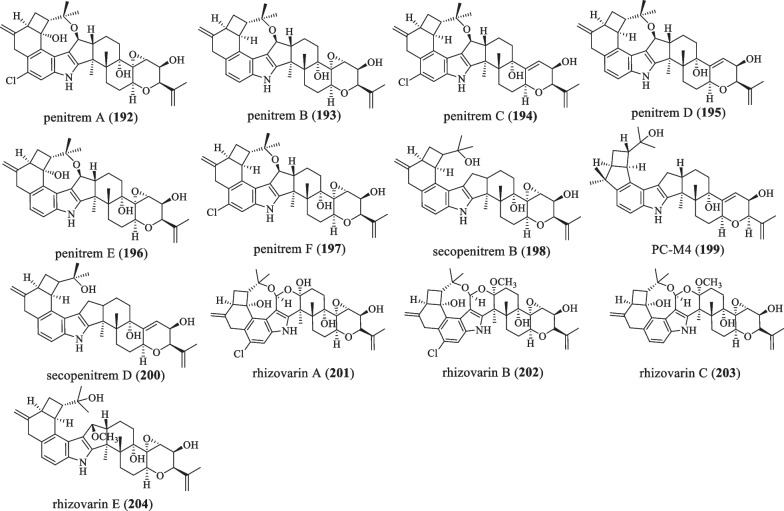
Chemical structures of paspaline-type compounds with A-ring 4/5 or 6 membered ring

The difference between this type and the last type is that the oxidative cyclization is modified into a 4/5 or 4/6-membered ring, and even further forms an oxygen-containing 8-membered ring with the 17th position of the **C** ring (Fig. 13 and Table 13). In 1983, Amelia et al. isolated 6 IDTs penitrems A–F (**192**–**197**) from *Penicillium* *crustosum*, wherein the compounds **192**, **194**, and **197** showed the anti-cancer effect on human A-549 and HL-60 cancer cell lines [49, 102]. It was also found that all chlorinated compounds (**192**, **194**, and **197**) exhibited more vigorous activity than their chlorine-free analogs, **193**, **195**, and **196** [24, 49]. In 1992, the compound secopenitrem B (**198**) was isolated from *Aspergillus* *sulphureus*, which was active against *H.* *zea* but inactive against *C.* *hemipterus*. It reduced weight gain by 87.0%, while **198** also caused 32.0% larval mortality [44]. In 1993, Yamaguchi et al. isolated PC-M4 (**199**) from *P.* *crustosum*, which could be biosynthesized by PC-M6, and then added isoprenyl to give PC-M5, which had no cancer cell activity [59]. In 2011, the compound secopenitrem D (**200**) was isolated and characterized from *P.* *crustosum*, which caused poisoning in animals [103]. In 2016, Gao et al. discovered the compounds rhizovarins A–C (**201**–**203**) and E (**204**) from Rhizomucor *Mucor* *irregularis* QEN-189, which represent the most complex members of the IDT derivatives. Among them, **201** and **202** showed activity against human A-549 and HL-60 cancer cell lines, and compound **204** showed activity against the A-549 cancer cell line, but not the Hela cell line [49].

**Table 13:** Name, bioactivities and source of compounds with A-ring 4/5 or 6 membered ring

Number	Compound name	Biological activity	Species origin	References
**192**	Penitrem A	Cancer cell activity	*P.* *crustosum*	[49]
**193**	Penitrem B	Anti-proliferative, anti-migration	*P.* *crustosum*	[49]
**194**	Penitrem C	Cancer cell activity	*P.* *crustosum*	[49]
**194**	Penitrem D	Anti-proliferative, anti-migration	*P.* *crustosum*	[49]
**196**	Penitrem E	Anti-proliferative, anti-migration	*P.* *crustosum*	[49]
**197**	Penitrem F	Cancer cell activity	*P.* *crustosum*	[49]
**198**	Secopenitrem B	Insect resistance	*A.sulphureus*	[44]
**199**	PC-M4		*P.* *crustosum*	[59]
**200**	Secopenitrem D	Poisons mammals	*P.* *crustosum*	[103]
**201**	Rhizovarin A	Cytotoxicity	*Mucor* *irregularis*	[49]
**202**	Rhizovarin B	Cytotoxicity	*Mucor* *irregularis*	[49]
**203**	Rhizovarin C		*Mucor* *irregularis*	[49]
**204**	Rhizovarin E	Cytotoxicity	*Mucor* *irregularis*	[49]

## Conclusion

This paper reviews the chemical structures of IDTs and their derivatives discovered in the past 50 years. Based on previous classifications, we divided 77 non-paspaline compounds into 6 categories according to their structural characteristics, and 127 paspaline-type compounds are divided into 7 categories according to oxidative modification. This provides convenient data for the future discovery of new compounds with similar structures or different oxidative modifications. At the same time, we also summarize the biophysiological activities of these compounds and their strong applications in pharmaceutical and agricultural markets. This also shows more compounds and provides more potent options for the development summary of future market applications.
